# Editorial: Reviews in cancer metabolism: 2023

**DOI:** 10.3389/fonc.2024.1478011

**Published:** 2024-09-24

**Authors:** Bela Ozsvari, Nadia Jacobo-Herrera

**Affiliations:** ^1^ Lunella Biotech Inc., Ottawa, ON, Canada; ^2^ Translational Medicine, School of Science, Engineering and the Environment, University of Salford, Manchester, United Kingdom; ^3^ Unidad de Bioquímica Guillermo Soberón Acevedo, Instituto Nacional de Ciencias Médicas y Nutrición Salvador Zubirán, Mexico City, Mexico

**Keywords:** cancer metabolism, metabolic therapy, metabolic reprogramming, resistance mechanisms, lactate metabolism, cancer stem cells

Cancer cells undergo distinct metabolic alterations to sustain their rapid growth and proliferation, separate from normal cells. This metabolic reprogramming, known as the Warburg effect, includes heightened aerobic glycolysis and glutamine metabolism, among other changes. Recent research has illuminated the intricate nature of cancer metabolism, showcasing varied metabolic characteristics among different types of cancer. Furthermore, new studies have emphasized the significance of metabolic interactions between cancer cells and the tumor microenvironment in advancing tumor growth and spread. Targeting these metabolic weaknesses has emerged as a promising approach in cancer therapy. Advances in metabolomics technologies have facilitated detailed analysis of cancer metabolites, offering valuable insights into the metabolic changes that fuel tumorigenesis. By unraveling the complexities of cancer metabolism, scientists strive to develop novel therapies that target specific metabolic pathways to enhance patient outcomes.

The reviews published in these Research Topic recapitulate the latest findings regarding lipid, iron, lactate metabolisms, ferroptosis, mitochondria-dependent metabolic reprogramming, and their potential in chemoresistance.

## Reprogramming of lipid metabolism in head and neck cancer

Dysregulation of lipid metabolism has recently emerged as a hallmark of cancer (1), as well as alterations of the lipid profile in head and neck cancer. Liang et al. described in their review on lipid metabolism reprogramming and its potential therapeutic targets in head and neck cancer (Liang et al.). Figure 1 demonstrates the most important enzymes involved in lipid metabolism in head and neck cancer showing the elevated level of uptake and synthesis of various fatty acids and sterol lipids in cancer cells. 2 lists the potential lipid metabolism-related therapeutic targets, mostly overexpressed enzymes of different fatty acid synthases. They also highlighted the deteriorating effects of prolonged consumption of tobacco, alcohol, and high-fat diets on healthy cells.

## Bibliometric analysis of NSCLC studies

Non-small cell lung cancer (NSCLC) accounts for most lung cancer histological types, which mainly include squamous cell carcinoma and adenocarcinoma, and a poor survival rate. Yang et al. in their review show a study where they collected and analyzed all the NSCLC and cancer metabolism-related research articles (2,246 in total) between 2013 and 2023 (Yang et al.). In their bibliometric analysis, authors retrieved articles from the Web of Science Core Collection (WoSCC) and the Bibliometrix packages (based on R), VOSviewer, and CiteSpace software to analyze numbers of articles, authors, countries/regions, institutions, references, journals, and keywords in the NSCLC-Met field. From this work, scientists can better understand research hot spots, new directions, and future development trends in the NSCLC-Met field.

## Role of AhR receptor in tumor resistance

Wang et al. detail in their review about Aryl hydrocarbon receptor (AhR), which is a ligand-activated nuclear transcription factor. AhR overexpression helps tumor cells evade the immune system by sending inhibitory signals to the immune cells through the tumor microenvironment (2). Figure 1 highlights the clinical applications of metabolic reprogramming in tumors. In Figure 2, the authors revealed that AhR elevation correlates with increased glycolysis in cancer cells. In conclusion, they suggest that AhR is crucial in the regulation of cellular metabolism, especially in tumor metabolic reprogramming.

## Iron metabolism and ferroptosis in cancer stem cells

Challenges in cancer treatment are metastasis, chemoresistance, and disease relapse; cancer stem cells (CSC) are known to be related to these phenomena. Wang et al. reviewed the impact of studying the cancer stem cells (CSCs) for cancer treatment (Wang et al.). CSCs can renew themselves, differentiate, and form new tumors, characteristics related to drug resistance, recurrence, and the spread of cancer cells to other parts of the body. Thus, targeting CSCs presents an opportunity for cancer treatment. They also delved into the changes observed in iron metabolism, lipid peroxidation, and the removal of lipid peroxides in CSCs, exploring their implications on ferroptosis. This research investigates the mechanisms governing iron metabolism and ferroptosis regulation in CSCs, extending the discussion to potential treatment tactics and new compounds that target CSCs by promoting ferroptosis.

## Abnormal lactate metabolism

It is well-documented how tumors satisfy their energy, biosynthesis, and redox requirements by undergoing metabolic reprogramming, resulting in an increased lactate level and other metabolites in the tumor microenvironment. According to Xu et al., lactate and lactylation mediate the reprogramming of immune cells and cellular adaptability to enhanced immunosuppression within the tumor microenvironment in hepatocellular carcinoma (HCC) (Xu et al.). The alteration of glucose metabolism and the Warburg effect in HCC leads to significant lactate production and accumulation, suggesting abnormal lactate modification in tumor tissue. In this context, Xu et al. reviewed the immune regulation of atypical lactate metabolism and lactate modification in hepatocellular carcinoma and the therapeutic approach of lactate-immunotherapy targeting, aiming to improve guidance for medication and treatment of patients with hepatocellular carcinoma.

## Chemoresistance and mitochondria-dependent metabolic reprogramming

One of the main problems of monotherapies is chemoresistance. A strategy to target different hallmarks of the cancer cell simultaneously could be an appropriate approach to stop drug resistance, metastasis, and disease recurrence. In leukemia, despite the efforts in drug development, chemoresistance is still of concern with the current chemotherapies, reducing the success of a complete recuperation, especially in elderly patients. Feng et al., introduced in their review the term “mitotherapy” and emphasized the importance of disordered mitochondrial metabolism and metabolic reprogramming as a therapeutic strategy in leukemia treatment, particularly in addressing chemoresistance (Feng et al.).

In summary, the reviews within this Research Topic discussed some metabolic alterations observed in cancer cells, highlighting the significance of understanding cancer metabolism for developing targeted therapies and improving patient outcomes. Studies on lipid, iron, and lactate metabolism, and their roles in chemoresistance, as well as the exploration of mitochondria-dependent metabolic reprogramming in addressing chemoresistance, offer valuable insights into potential treatment strategies. By targeting specific metabolic pathways and identifying metabolic vulnerabilities, there is potential for innovative therapeutic approaches in cancer treatment.
